# Genetic diversity of a widespread annual killifish from coastal Tanzania

**DOI:** 10.1186/s12862-019-1549-2

**Published:** 2020-01-06

**Authors:** Veronika Bartáková, Béla Nagy, Matej Polačik, Radim Blažek, Hieromin Lamtane, Martin Reichard

**Affiliations:** 1 Czech Academy of Sciences, Institute of Vertebrate Biology, Květná 8, 603 65 Brno, Czech Republic; 2 Fontainebleau, France; 3 Department of Animal, Aquaculture and Range Sciences, Sokoine University of Agriculture, P.O. Box 3004, Morogoro, Tanzania

**Keywords:** Dispersal, Eastern Africa, River morphology, Temporary pool, mtDNA, Historical demography

## Abstract

**Background:**

African annual killifishes (*Nothobranchius* spp.) are adapted to seasonally desiccating habitats (ephemeral pools), surviving dry periods as dormant eggs. Given their peculiar life history, geographic aspects of their diversity uniquely combine patterns typical for freshwater taxa (river basin structure and elevation gradient) and terrestrial animals (rivers acting as major dispersal barriers). However, our current knowledge on fine-scale inter-specific and intra-specific genetic diversity of African annual fish is limited to a single, particularly dry region of their distribution (subtropical Mozambique). Using a widespread annual killifish from coastal Tanzania and Kenya, we tested whether the same pattern of genetic divergence pertains to a wet equatorial region in the centre of *Nothobranchius* distribution.

**Results:**

In populations of *Nothobranchius melanospilus* species group across its range, we genotyped a part of mitochondrial cytochrome oxidase subunit 1 (*COI*) gene (83 individuals from 22 populations) and 10 nuclear microsatellite markers (251 individuals from 16 populations). We found five lineages with a clear phylogeographic structure but frequent secondary contact. Mitochondrial lineages were largely congruent with main population genetic clusters identified on microsatellite markers. In the upper Wami basin, populations are isolated as a putative *Nothobranchius prognathus*, but include also a population from a periphery of the middle Ruvu basin. Other four lineages (including putative *Nothobranchius kwalensis*) coexisted in secondary contact zones, but possessed clear spatial pattern. Main river channels did not form apparent barriers to dispersal. The most widespread lineage had strong signal of recent population expansion.

**Conclusions:**

We conclude that dispersal of a *Nothobranchius* species from a wet part of the genus distribution (tropical lowland) is not constrained by main river channels and closely related lineages frequently coexist in secondary contact zones. We also demonstrate contemporary connection between the Ruvu and Rufiji river basins. Our data do not provide genetic support for existence of recently described cryptic species from *N. melanospilus* complex, but cannot resolve this issue.

## Background

Ephemeral habitats with patchy distribution are common but represent challenging environment to dispersal and colonization. While some species have evolved specialised stage to overcome dispersal limitation, others have responded by strong population resilience and possess stages resistant to shifting environmental conditions. For example, small cladocerans often produce resting stages that are dispersed by wind and, consequently, many cladoceran species have very large ranges and weak genetic structure (e.g. [1]). In contrast, stream gammarids (Amphipoda) are poor dispersers but resistant to temporary environmental challenges, and evolved into locally endemic lineages and species (e.g. [2]).

Temporary freshwater pools are common in highly seasonal environments, including African savanna. Their specialised fauna includes widespread invertebrate species with specific dispersal stage (e.g. crustaceans and aquatic insects) as well as specialised killifish species that are extremely poor dispersers [3, 4]. In African savanna, ephemeral pools inhabited by fishes vary in their temporal dynamics, from exclusively rain-fed pools of a brief existence (< 1 month) in semi-arid Mozambican savanna to semi-permanent networks of pools within active river alluvia in equatorial regions [5]. However, nothing is known on how such contrasting conditions affects connectivity of populations and their genetic diversity and phylogeographic structure.

Annual killifish are adapted to ephemeral pools in Africa and Neotropics by possessing a resilient developmental stage. After habitat desiccation, their embryos survive in dry pool sediment in the form of diapausing eggs [6, 7]. Within the order Cyprinodontiformes (killifishes, toothcarps and livebearers) annual life history evolved at least six times [8], with habitat desiccation often becoming obligatory for successful embryo development [9]. In Eastern Africa, over 85 recognized species of the genus *Nothobranchius* inhabit ephemeral freshwater pools developed on vertisol (dark cracking clay) soils [5]. *Nothobranchius* distribution covers extensive region from relatively dry subtropical areas with a single rainy season in the north and south across humid equatorial areas with two rainy seasons a year. *Nothobranchius* diversification follows allopatric scenario [10], with isolating populations through separations of drainages through landscape faulting and warping [11, 12]. The same mode of diversification apparently pertains to intra-specific level. The dry southern part of *Nothobranchius* range (Mozambique) harbours highly geographically structured populations, with important roles of genetic drift and dispersal limitation [13–15]. In that region, major rivers formed significant barriers to killifish dispersal, leading to suture zones shared by coexisting *Nothobranchius* lineages [15]. In addition, intra-specific variation is structured along elevational gradient [15]. Such phylogeographic pattern is exceptional as it combines features of both aquatic and terrestrial taxa.

In the present study, we tested whether the patterns of *Nothobranchius* genetic diversity from dry subtropical region pertain to wet equatorial region of African savanna. Specifically, we investigated population genetic pattern in *Nothobranchius melanospilus* species group, geographically widespread and locally common killifish in lowland East Africa [16]. This species group contains *Nothobranchius melanospilus* (Pfeffer, 1896) and two recently described cryptic species; *Nothobranchius prognathus* Costa 2019 and *Nothobranchius kwalensis* Costa 2019 from peripheral parts of the *N. melanospilus* range [17]. The species group inhabits large lowland region of southeastern Kenya and eastern Tanzania, an important hotspot of biological diversity with a high occurrence of endemic species [18, 19], including notable diversity of *Nothobranchius* fishes [16]. Using 264 individuals from 22 populations, we combined information from mitochondrial cytochrome oxidase subunit 1 (*COI*) gene and 10 nuclear microsatellite markers to examine the role of river basins, river channels and elevational gradient in structuring *N. melanospilus* species group.

## Methods

### Study taxon and study area

*Nothobranchius melanospilus* is the most commonly recorded species of the genus. It is found in natural temporary pools, swamps and small temporary streams as well as in ricefields, man-made ditches and culverts [16]. The species group has wide geographical distribution and its populations are common across coastal area (3 m above sea level) up to elevation of 425–490 m in the upper Wami basin (Tendigo swamp) [16, 17]. Geographically, populations are recorded from the Umba and Ramisi basins in the southeastern tip of Kenya, across large region of coastal Tanzania (Pangani, Wami, Ruvu, Mbezi, Ruhoi and Rufiji basins) and from the island of Zanzibar off the Ruvu river [16, 20, 21].

A recent taxonomic work used museum specimens [17] to formally describe existence of two previously unrecognised species within *N. melanospilus*, on the basis of combination of morphometric characters and female colouration. Based on that study [17], *N. melanospilus* sensu stricto is distributed only south of the Wami basin (and on Zanzibar Island). The upper Wami basin populations were described as *Nothobranchius prognathus* Costa 2019 and populations from southeastern Kenya as *Nothobranchius kwalensis* Costa 2019. Hence, our study putatively concerns to a complex of three closely related species of the *N. melanospilus* group. Our samples cover the entire range of the species group, except for Zanzibar Island and the lower Pangani basin.

The region of *N. melanospilus* species group distribution was modified by East African Rift tectonics that formed the Ruvu and Rufiji throughs [5]. The two basins share a common swampy area in their middle and upper reaches. Island of Zanzibar, also inhabited by *N. melanospilus* species, is located on a shallow shelf near the mouths of the Ruvu and Wami rivers and was likely linked to mainland populations until 12,000 years ago. Small coastal rivers between the Ruvu and Rufiji (e.g. the Luhule and Mbezi rivers) are separated from the Ruvu and Rufiji by Pugu and Mtoti hills, forming so-called Mbezi Triangle with endemic *Nothobranchius* species [5, 16]. The lower reaches of Wami and Pangani and a small Ramisi river share a low-lying coastal strip, while the upper Wami basin (Tendigo swamp) has limited connection to the floodplain pools of the lower Wami basin [5].

### Sampling and genotyping

Specimens from most populations were collected during a dedicated field trip in May and June 2017, using dip and seine nets. Fish were identified in the field; unlike in other Tanzanian *Nothobranchius*, female *N. melanospilus* species group are readily recognised from other *Nothobranchius* species by their unique dark spots on the body [16]. Most fish were identified on the bank, small fin clips were taken from their caudal fin and stored in 98% ethanol. Fish were then released back to their habitat. Voucher specimens (a random subsample of both sexes) were taken from most populations and are stored at the Institute of Vertebrate Biology, Brno, Czech Republic. Sixteen specimens from 7 populations were collected by B.N. (Table 1), using a similar method. This included a sample of putative *N. kwalensis* from the Ramisi basin (4 individuals from 2 populations). All field sampling and export procedures followed regulations of Tanzania, with permits and research associateship issued by Sokoine University of Agriculture in Morogoro (research permit: RPGS/R/AS/11/2017; export permit AS/A/1).

**Table 1:** Overview of analysed populations, with their collection code (Population ID), GPS coordinates, identification of river basin, elevation (in m above sea level), number of individuals analysed on 10 microsatellite markers (N_MS_) and on mitochondrial COI sequence (N_COI_), and assignment of individuals to one of five haplogroups (Haplogroup). Populations are ranked by their elevation within river basins

Population ID	GPS_S	GPS_E	Basin	Elevation	Habitat type	N_MS_	N_COI_	Haplogroup
T15	6.63624	38.16554	Ruvu	246	Isolated pool	21	4	Wami
T14	6.86204	38.18471	Ruvu	161	Ephemeral stream	17	4	Ruvu
T62	6.60366	38.33852	Ruvu	166	Pool	2	3	Ruvu
TZN 09–2^a^	6.69247	38.75305	Ruvu	62	Pool	0	3	Ruvu
T57	6.69268	38.75316	Ruvu	62	Pool	27	4	Ruvu
T02	6.70380	38.67541	Ruvu	22	Pool	29	3	Ruvu
T64	6.46973	38.79884	Ruvu	21	Man-made pools	19	4	Ruvu
TZN 09–1	6.46063	38.90732	Ruvu	19	Pool	0	3	Ruvu/Mbezi
T51	6.45595	38.90742	Ruvu	17	Rice field	3	3	Ruvu
T50	6.51363	38.95730	Ruvu	14	Pool	9	6	Mbezi
TZN 17–9^a^	6.47548	38.85812	Ruvu	4	Floodplain pool	12	3	Ruvu
T17	8.12097	38.96849	Rufiji	50	Pool	9	6	Ruvu
TZN 18–2^a^	8.10159	38.99509	Rufiji	30	Pool	0	1	Rufiji
T16	8.07289	38.98788	Rufiji	23	Culvert by main road	18	7	Rufiji/Ruvu
TZN 17–1^a^	8.05565	38.98293	Rufiji	20	Pool	0	2	Rufiji/Ruvu
T31	7.19349	39.17192	Mbezi	65	Deep pool with rice field	22	4	Mbezi
T35	7.35934	39.12495	Mbezi	31	Rice field	20	8	Ruvu
KEN 15–1^a^	4.52267	39.29908	Ramisi	21	Floodplain pool	0	1	Ramisi
KEN 08–23^a^	4.51842	39.29303	Ramisi	21	Pool	0	3	Ruvu
T06	6.59145	37.59217	Wami	435	Swamp	21	4	Wami
T09	6.72178	37.12161	Wami	425	Pool in swampy area	19	4	Wami
T83	6.76608	37.16220	Wami	425	Pool	3	3	Wami

^a^samples collected by Béla Nagy

In the laboratory, DNA was extracted using the DNeasy Blood and Tissue Kit (Qiagen) following a standard protocol. Full details of the genotyping methods, primer sequences, microsatellite multiplexing, and PCR protocols are presented in Additional file 1. In brief, partial mitochondrial *COI* gene was amplified using primers TRNYF1 (AGG GAG TTA CAA TCC ACC ACT ATT T) and TRNSR1 (ATG GGG GTT CAA TTC CTT CCT TT), alternatively, and a forward primer COI852F (CTT TAT TGT TTG AGC CCA CCA CA) [12] for a set of 83 individuals from 22 populations (Table 1). PCR products were sequenced commercially in Macrogen and GATC Biogen. All sequences have been deposited in GenBank (accession numbers MN413245–MN413327). Initially, we aimed to genotype partial cytochrome *b* gene, but none of the 7 tested primers (Additional file 1) amplified successfully.

We used a set of 10 microsatellite loci in four multiplex PCR sets (for details see Additional file 1) to genotype a sample of the 251 individuals from 16 populations (Table 1). PCR products were separated on the ABI Prism® 3130 Genetic Analyzer (Applied Biosystems) and analysed using GeneMapper® v. 3.7 (Applied Biosystems).

### Analysis of mitochondrial DNA variation and historical demography

Phylogenetic relationships within *COI* dataset were inferred by Bayesian (BI) approach. We used PartitionFinder v. 2.1.1 [22] to select the most suitable substitution models for different parts of mtDNA using the corrected Akaike Information Criterion (HKY, GTR, and SYM + I for individual positions in codon). One sequence of *Nothobranchius guentheri* was used as an outgroup. Bayesian analysis was performed by Markov Chain Monte Carlo (MCMC) simulation using MrBayes 3.2.6 [23]. Two independent analyses were initiated from random trees. Three heated and one cold chain were run for 20 million generations per run, sampling every 1000 generations, and 25% of trees were discarded as burn-in. Bayesian posterior probabilities were used to evaluate branch support of the tree. Phylogenetic analysis was performed on Cipres Science Gateway webserver [24] and the final tree was edited in FigTree v1.3.1 (http://tree.bio.ed. ac.uk/software/figtree).

All sequences were geo-referenced and the geographical distribution of lineages was plotted onto map using QGIS 2.18 (http://qgis.org). Diversity estimates, i.e. number of polymorphic sites (*Np*), number of haplotypes (*Nh*), haplotype diversity (*Hd*), nucleotide diversity (π, expressed as percentages, i.e. 0.001 = 0.1%), the average number of nucleotide differences (*k*) and Watterson’s estimate of θ (θ = 4Ne*μ) were calculated using DnaSP v. 5.10.01 [25].

Within two most widespread lineages, historical demography was estimated using the neutrality tests, Tajima’s D and Fu’s FS, sensitive to population size changes [26] in DnaSP [25], with significantly negative values reflecting recent population expansion. Ramos-Onzins and Rozas R2 tests were also computed due to relatively lower sample size, with *P*-values obtained by coalescent simulations with 10,000 replicates in DnaSP [25]. Additionally, the distribution of pairwise nucleotide differences (mismatch distribution; MD) was calculated in DnaSP. We used the sum of square deviations (SSD) between the observed and expected mismatch as a test statistic for the validity of the estimated stepwise expansion model [27]. Parameter τ (the moment estimator of time to the expansion) was estimated with DnaSP using the moment method of Rogers [28] assuming the infinite sites model (IFM) and, additionally, in ARLEQUIN [29] using the method of Schneider and Excoffier [27] to relax the IFM assumption. Confidence intervals were obtained by a parametric bootstrap approach based on 1000 replicates performed in ARLEQUIN [29].

### Intra-population analysis of microsatellite marker variation

The proportion of null alleles (NA) at each locus and population was estimated in FreeNA [30]. The mean frequency of microsatellite null alleles per population was greater than 5% for five loci (Additional file 1). The greatest proportion of null alleles was 13.6% for Nfu_0027_FLI locus.

Deviations from linkage and Hardy-Weinberg equilibrium (HWE) for each locus and population were detected in Genepop 4.0.10 [31, 32]. Linkage disequilibrium among 10 microsatellite loci and HWE (“Exact probability test”) were tested using Markov chain methods (dememorization: 10,000, batches: 100, iterations per batch: 5000). Correction for multiple testing was performed using false discovery rate approach (FDR) in QVALUE [33]. Only 5 out of 441 pair-wise results of genotypic linkage disequilibrium tests were significant at *p* < 0.05. Pairs of loci were significantly linked only in one or two populations and the microsatellite loci can be considered to be unlinked.

Genetic variability was estimated by calculating observed heterozygosity (H_O_) and unbiased expected heterozygosity according to Nei (1978) (H_E_) in GENETIX 4.05.2 [34]. Mean allelic richness (AR) was determined with the rarefaction method in FSTAT 2.9.3 [35] to estimate the expected number of alleles standardized to the smallest population sample of 8 individuals. Pairwise genetic differentiation was calculated with GENETIX 4.05.2. For analysis of genetic variability (HWE, H_O_, H_E_, AR) populations with < 8 sampled individuals were not used (thus excluding populations T51, T62, and T83).

### Inter-population analysis of genetic structure

To quantify genetic differentiation between populations, we computed pairwise estimators of *F*_ST_ for each pair of populations using the ENA correction described in [30] and implemented in the software FreeNA [30], as there was some evidence of null alleles. We then used these corrected values to test for isolation-by-distance pattern by regressing pairwise estimates of *F*_ST_/(1- *F*_ST_) against ln-distance between sample sites. Mantel tests were used to test the correlation between matrices of genetic differentiation and geographical distances between sampling sites by 1000 permutations in Genepop 4.0.10 [32].

To investigate the spatial genetic structure among individuals, we used STRUCTURE 2.3.4 [36]. The individual-based Bayesian clustering procedure was run with 20 independent runs for each of K from 1 to 10. Each run included 10^6^ iterations, following a burn-in period of 10^5^ iterations. We used admixture ancestry model and correlated allele frequencies model (with λ = 1). The output of STRUCTURE analysis was post-processed in CLUMPAK software [37] to identify separate groups of runs on the base of similarity between Q-matrices for each K. We used the LargeKGreedy algorithm, random input order and 2000 repeats. Different modes from the results of the 20 runs for each K value at a threshold of 0.9 for similarity scores were identified. Summary barplots for a given K value contain averaged proportions of individual membership obtained for all runs in the same mode. The likelihood of K (Ln Pr(X|K)), the ΔK criterion [38] and a proportion of similar runs that formed the major modes for each K were used to infer the best number of real populations.

## Results

### Mitochondrial lineages: distribution and demographic history

**Fig. 1:**
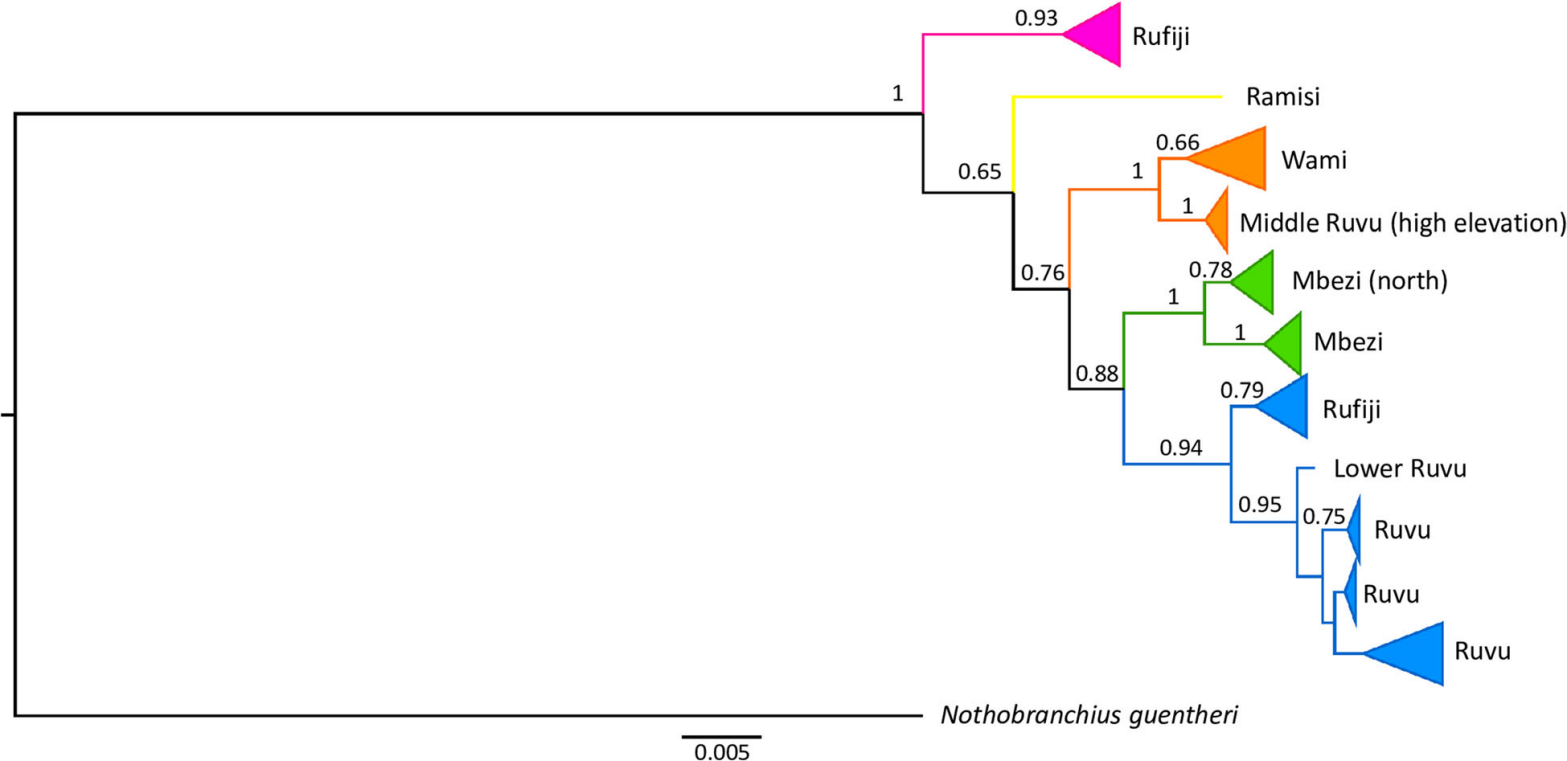
Bayesian reconstruction of mitochondrial phylogeny of the *N. melanospilus* species complex based on 83 *COI* sequences (657 bp). Bayesian inference posterior probabilities (MrBayes 3.2.6) are shown for each nod

**Fig. 2:**
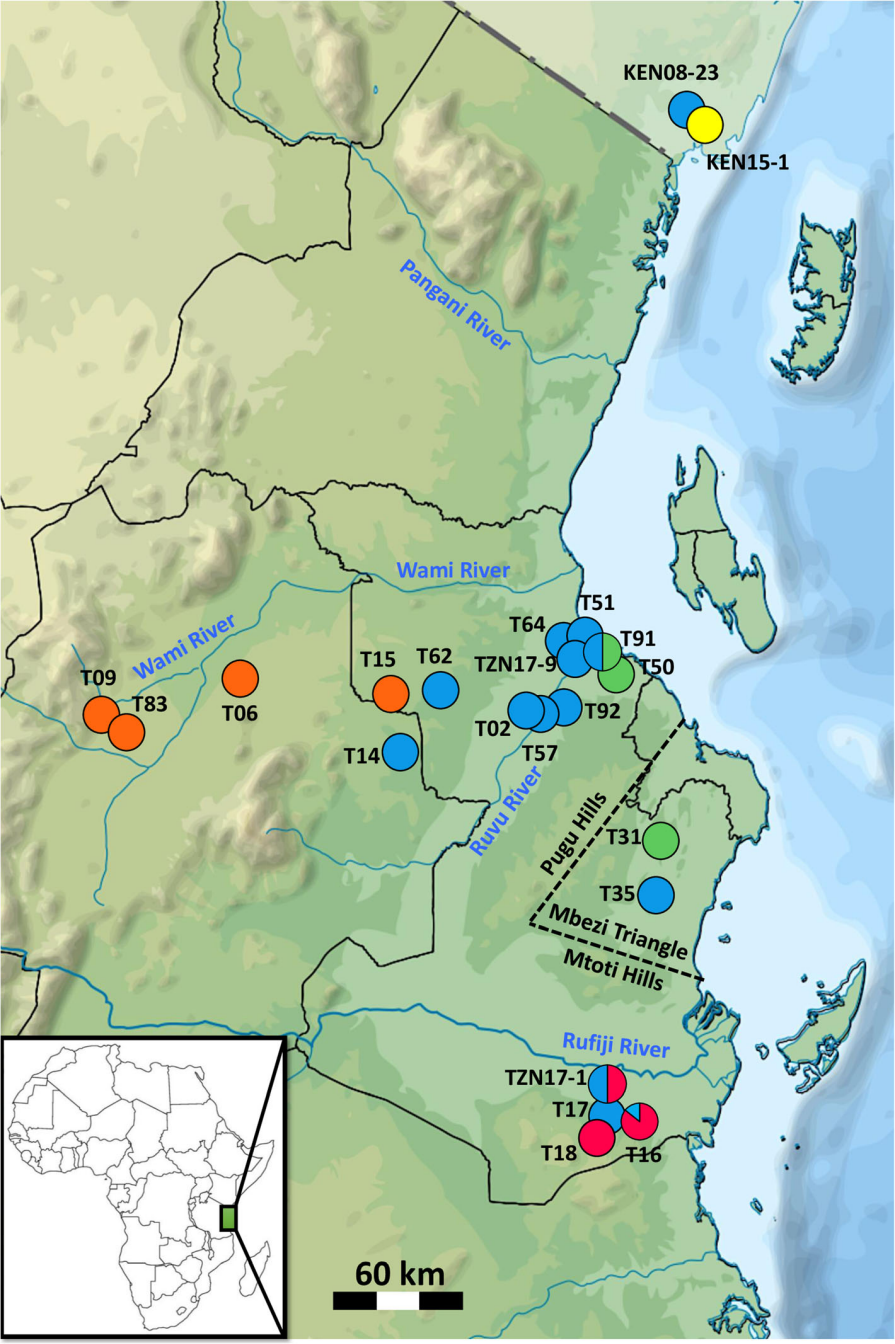
Geographic distribution of *Nothobranchius melanospilus-*species group mitochondrial lineages. The colours correspond to the lineages as defined in Fig. 1 and indicate the relative proportions of lineages at a particular locality. Names of localities correspond to those in Table 1. The map has been modified from open-access source map that is free to re-use and adapt under CC-BY-SA-3.0 licence and is available at https://commons.wikimedia.org/wiki/File:Tanzania_relief_location_map.sv

Among 22 populations (83 individuals), we detected 30 different haplotypes of *COI* sequences (657 bp). Their phylogenetic analysis revealed five main lineages, some of which possessed a finer substructure (Fig. 1, Additional file 2). The lineages had clear geographic structure but common contact zones (Fig. 2) and generally received low statistical support (Fig. 1). Only the basal lineage (Rufiji, red in Fig. 2) had strong node support (Fig. 1). This lineage was found exclusively in the Rufiji basin, in the south of the species range, where it widely coexisted with the second lineage (Ruvu, blue). The Ruvu lineage was most common and geographically most widespread. It was dominant in the middle and lower Ruvu basin, but found across the north-south axis of the species range, from coastal Kenya to the Rufiji basin, including Mbezi Triangle. Small coastal basins of Mkuza and Mbezi harboured individuals from the third lineage (Mbezi, green) that locally coexisted with the Ruvu lineage in the lower Ruvu basin (population T91). The putative *N. prognathus* was represented by the fourth lineage (Wami, orange), from pools within the upper Wami River basin (Tendigo swamp). In addition, Wami lineage pertained to one pool in an isolated part of the middle Ruvu basin (population T15). This haplogroup was distributed at the highest elevation (246–435 masl compared to < 167 masl in other lineages), but had low statistical support (BI = 0.76, Fig. 1). Finally, a single individual with a unique haplotype (Ramisi, yellow lineage) was found in coastal Kenya (population KEN08–23), perhaps the putative *Nothobranchius kwalensis*. More specimens were not available from that population. The distinctness of this haplotype had low statistical support (BI = 0.65, Fig. 1), though we note that our phylogenetic inference is based only on a fragment of 657 bp. Interestingly, all three individuals from the adjacent population (KEN15–1, located only 15 km from KEN08–23) possessed haplotypes of the common Ruvu lineage (Fig. 2).

Demographic history was analysed separately for geographically two most widespread mitochondrial lineages – Ruvu (blue) and Wami (orange). Unimodal pattern of mismatch distribution graphs (Additional file 2) and Ramos-Onsins & Rozas’ R2 (Table 2) suggested recent population expansion of both haplogroups. The tests of neutrality (Tajima’s D and Fu’s FS) sensitive to sample size limitations demonstrated significant expansion only in the Ruvu haplogroup (Table 2). The sums of squared deviations (SSD) of the mismatch distribution were not significant, indicating that the curves support the sudden expansion model. Full details of *COI* variability and historical demography are shown in Table 2.

**Table 2:** Analysis of mitochondrial variability and historical demography. The number of sequences (N), number of haplotypes (h), haplotype (gene) diversity with one Standard Deviation (Hd), number of polymorphic (segregating) sites (S), nucleotide diversity (in %; with one Standard Deviation) (π), average number of nucleotide differences (k), Tajima’s D (with significance at *P* < 0.05 denoted by asterisk), Fu’s FS (with significance at *P* < 0.01 denoted by asterisk), Ramos-Onsins and Rozas R2 (with significance at *P* < 0.05 denoted by asterisk), onset of population expansion assuming the stepwise growth model (τ Arl, with 95% confidence interval), τ DnaSP (the moment estimator of time to the expansion), sum of squared deviations (SSD)and the probability of observing a less good fit between the model and the observed distribution by chance (P_SSD_) and the mismatch observed mean (ObsMean)

Haplogroup	N	h	Hd	S	π (%)	k	Tajima’s D	Fu’s FS	R2	τ Arl (95% CI)	τ DnaSP	SSD	P_SSD_	ObsMean
All pooled	83	30	0.858 ± 0.035	67	1.909 ± 0.147	12.525	–	–		**–**	–	–	–	–
Wami	15	9	0.886 ± 0.062	11	0.403 ± 0.057	2.648	−0.838	−3.30	0.050*	3.334 (0.842–5.777)	2.648	0.00887	0.589	2.648
Ruvu	44	11	0.593 ± 0.087	13	0.148 ± 0.038	0.973	−2.068*	−7.41*	0.101*	0.859 (0.391–1.563)	0.252	0.00268	0.545	0.973

Note that recently admixed populations were excluded from analyses

### Nuclear microsatellite markers: intra-population variability

Based on data from 10 microsatellite loci, most populations (nine out of 13) showed deviance from HWE, when calculated over all loci (Table 3). In most cases deviations from HWE were caused by null alleles present with an increased frequency at some loci and populations, probably as a result of the “ascertainment bias”.

**Table 3:** Measures of intra-population genetic variability based on analyses of microsatellite markers. Sample size (N), P-values of the Fisher’s exact test for deviation from Hardy-Weinberg equilibrium (HWE), expected heterozygosity based on Nei estimate (H_E_), observed heterozygosity (HO) and allelic richness estimated for 8 individuals using rarefaction (AR). Populations are ranked from highest AR

Population	Basin	N	HWE	H_E_ (Nei)	H_O_	AR
T14	Ruvu	17	< 0.001	0.8796 ± 0.0693	0.7381 ± 0.1881	8.615
T64	Ruvu	19	< 0.001	0.8580 ± 0.0916	0.7269 ± 0.2265	8.363
T02	Ruvu	29	< 0.001	0.8531 ± 0.0865	0.6823 ± 0.2008	8.235
TZN 17–9	Ruvu	12	< 0.001	0.8497 ± 0.1095	0.7203 ± 0.1847	8.181
T17	Rufiji	9	< 0.001	0.8458 ± 0.1071	0.6458 ± 0.3079	7.939
T16	Rufiji	18	< 0.001	0.8565 ± 0.0807	0.7233 ± 0.2086	7.934
T09	Wami	19	0.0217	0.7331 ± 0.3114	0.6737 ± 0.3391	7.819
T50	Ruvu	9	< 0.001	0.8437 ± 0.1040	0.5944 ± 0.3248	7.643
T35	Mbezi	20	< 0.001	0.8270 ± 0.1252	0.6611 ± 0.1788	7.260
T06	Wami	21	0.2836	0.6907 ± 0.3046	0.6652 ± 0.3290	6.504
T15	Ruvu	21	0.0083	0.6920 ± 0.1694	0.6375 ± 0.2992	5.162
T31	Mbezi	22	0.1695	0.6339 ± 0.2561	0.6089 ± 0.2904	4.716
T57	Ruvu	27	< 0.001	0.6785 ± 0.1736	0.5362 ± 0.2323	4.600

All measures of intra-population genetic variation (H_O_, H_E_, AR) for populations with at least 8 individuals are shown in Table 3. The range of AR was 4.60–8.62 (rarefaction estimate for the lowest sample size *N* = 8). The lowest intra-population genetic variability (H_E_ < 0.70, AR < 5.2) was detected in populations T57, T31 and T15 located at relatively isolated pools. In contrast, the highest intra-population genetic diversity (H_E_ ≥ 0.85, AR > 7.9) was found in populations from the floodplain of lower parts of the major rivers (Ruvu: TZN17–9, T64, T02, all below 22 masl; Rufiji: T16, T17, below 50 masl) and in population T14 in the middle reach of the Ruvu (Table 3).

### Nuclear microsatellite markers: genetic structure

We detected high level of genetic structuring among *N. melanospilus*-group populations, with the mean (± S.E.) pairwise *F*_ST_ = 0.113 ± 0.07. The pairwise *F*_ST_ values were significantly different from zero in 95.83% of population pairs; only five of 120 pairwise *F*_ST_ were not significant (Additional file 3). Four non-significant *F*_ST_ values were between pairs of geographically close populations, though one non-significant *F*_ST_ was between geographically distant populations from different basins (T14 and T17, from the Ruvu and Rufiji basins, respectively). The pattern of isolation-by-distance showed weak but significant association between geographical and genetic distances (Mantel test, 1000 permutations, *P* = 0.03, Additional file 2: Figure S2).

**Fig. 3:**
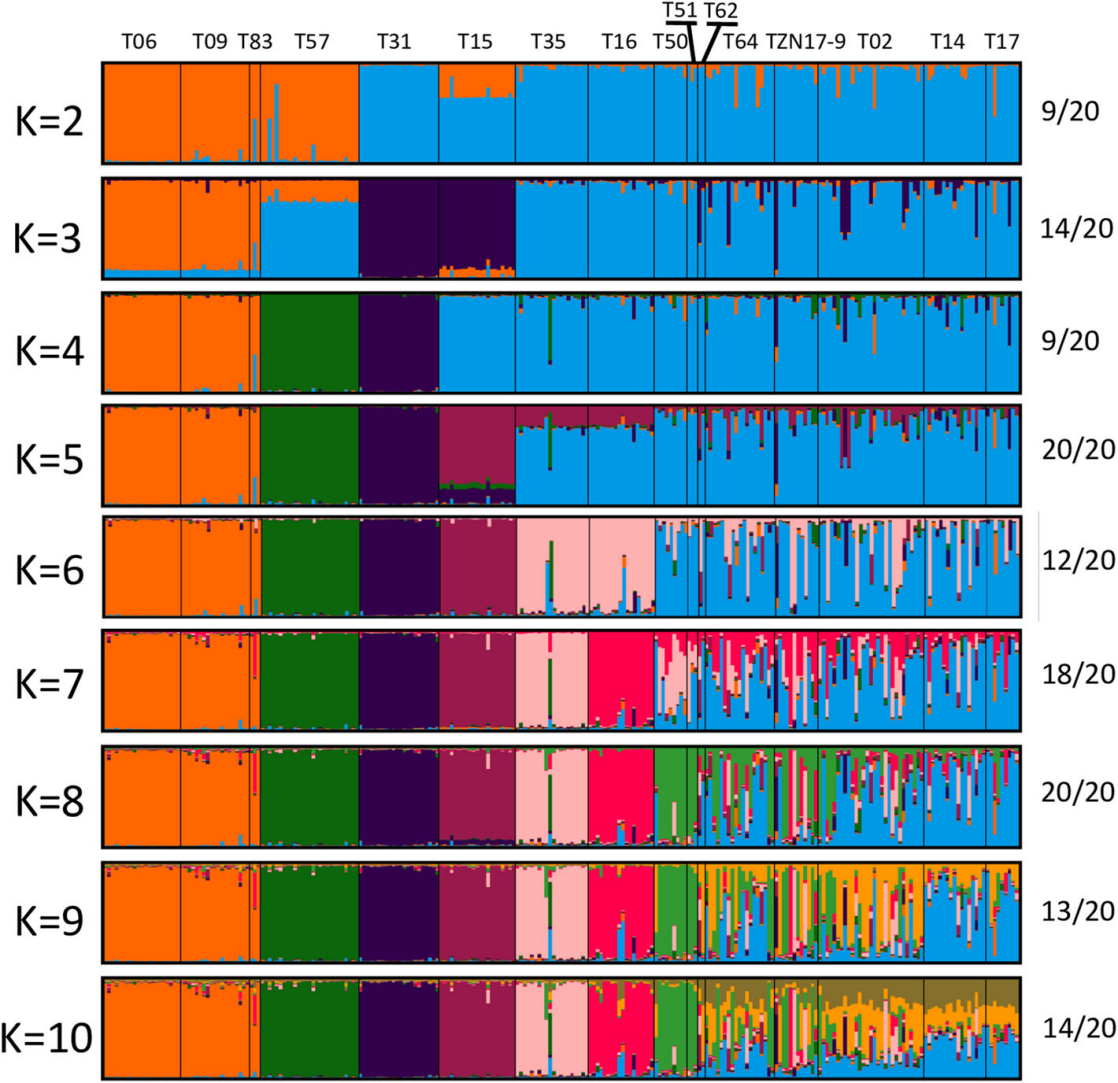
Bayesian analysis of genetic similarity among *Nothobranchius melanospilus-*species group populations performed in STRUCTURE for 251 individuals from 16 populations for *K* = 2–10

**Fig. 4:**
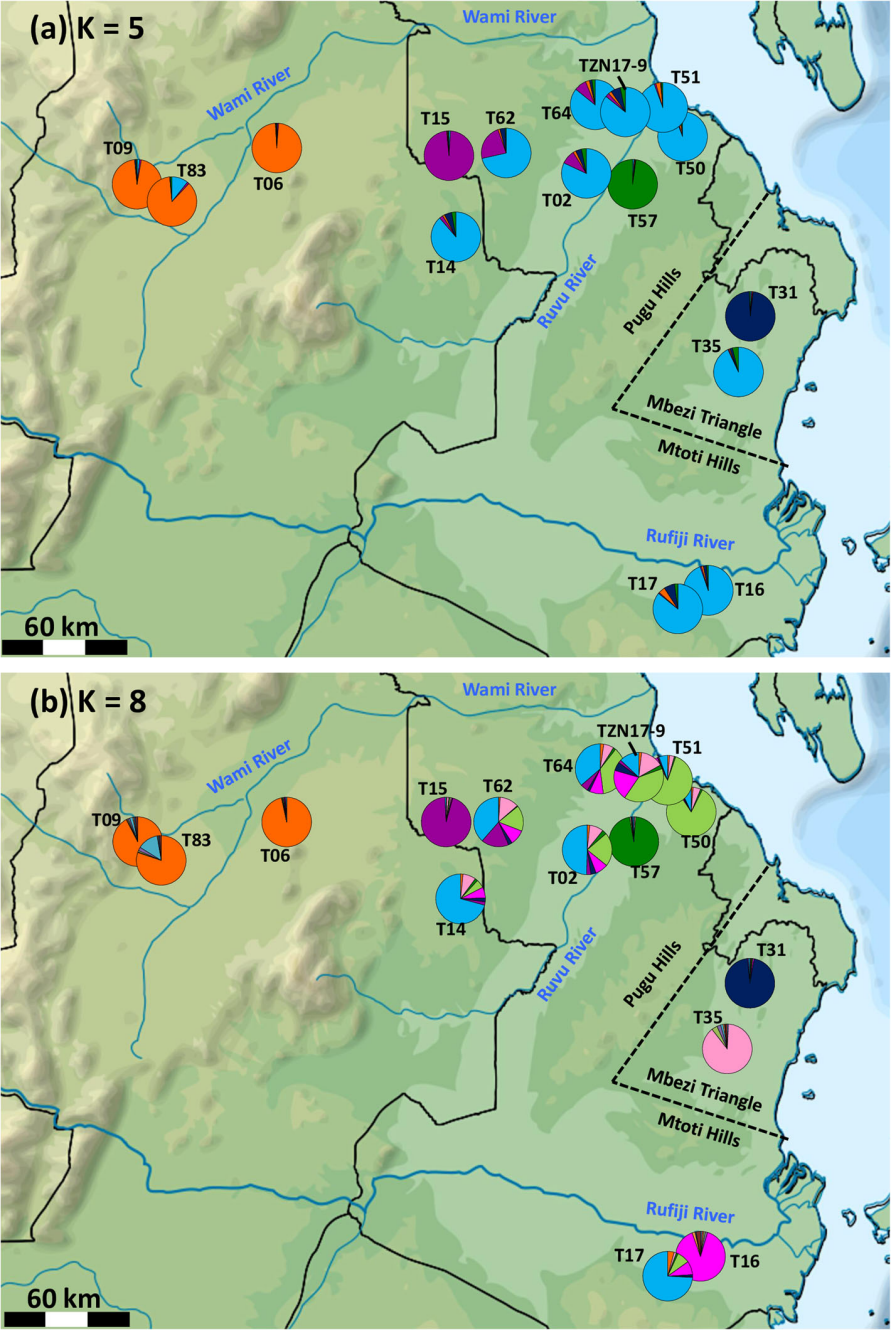
Geographic distribution of genetic diversity in *Nothobranchius melanospilus-*species group from nuclear microsatellites based on assignment to 8 clusters (**a**) and 5 clusters (**b**) following STRUCTURE analysis across study area. Pie chart colours represent the proportional membership of individuals to microsatellite-based clusters inferred from the models selected using the approach of Evanno et al. [38]. Names of localities correspond to those in Table 1. The map has been modified from open-access source map that is free to re-use and adapt under CC-BY-SA-3.0 licence and is available at https://commons.wikimedia.org/wiki/File:Tanzania_relief_location_map.svg

Using Bayesian clustering in STRUCTURE, the most suitable model to separate sampled populations was for K = 8, based on the likelihood of K (Ln Pr(X|K)), the ΔK criterion [38], and a proportion of similar runs (Additional file 4, Fig. 3). The Wami basin populations were consistently separated from all other populations (Fig. 3). Populations from the Ruvu and Rufiji basins, as well as populations from Mbezi Triangle were all clustered at lower K values, but increasing the number of assumed clusters led to the separation of two Mbezi populations (T31, T35) to reciprocally unique clusters (Fig. 3). Three individual populations were separated to unique clusters at K = 8; isolated populations T15 (from the Wami lineage) and T57 (Ruvu lineage) from the Ruvu basin (both with low intra-population variability), and T16 (one fish from the Ruvu and six fish from the Rufiji lineage) from the Rufiji basin (Fig. 4). Finally, one lower Ruvu (T51, Ruvu lineage) and one lower Mkuza (T50, Mbezi lineage) populations, both located very close to the coast clustered together at K = 8 (Fig. 4).

## Discussion

Populations of *Nothobranchius melanospilus* species group were clearly structured, with geographically adjacent lineages sometimes coexisting within a single population. Clustering to mitochondrial lineages had a good correspondence in nuclear microsatellite data. The most widespread lineage (Ruvu, blue) was found in all sampled regions except of the Wami basin. It coexisted with Rufiji (red) lineage in the lower Rufiji basin and Mbezi (green) lineage in the lower Ruvu. Its broad distribution agrees with a clear signature of recent expansion indicated by neutrality indices. Despite their coexistence, the three common lineages (Ruvu, Rufiji, Mbezi) had their apparent centres of distribution that are indicative of potential refugial persistence in the lower Ruvu basin, lower Rufiji basin and smaller coastal rivers between the Ruvu and Rufiji, including Mbezi Triangle.

Three upper Wami basin populations formed a separate cluster on mitochondrial and nuclear markers, apparently consistent with a recent elevation of the populations from this region to a specific level, as *N. prognathus* [17]. In our mitochondrial dataset, this lineage additionally included one population (T15) from an isolated pool at the periphery of the Ruvu basin, but this population did not cluster with the three Wami populations on nuclear markers. On nuclear markers, this population was recovered as relatively unique since it also differed from geographically close Ruvu population. The difference was likely driven by its low intra-population genetic diversity and hence important contribution of genetic drift. Indeed, that isolated population is located at relatively high elevation (246 masl; compared to ≤166 masl in all other non-Wami populations, and 425–435 masl in the Wami populations). Unlike for the other four lineages, we have not confirmed coexistence of the Wami lineage with any other haplogroups in the sample of populations we studied. Investigation of finer-scale population genetic pattern in that region could resolve separation of the Wami and Ruvu lineages and clarify whether *N. prognathus* should be considered as a valid species.

A single divergent haplotype has been discovered in coastal Kenya (Ramisi basin, KEN15–1). Validity of this haplotype has been confirmed by repeated analysis (including new DNA sampling from voucher specimen). Morphological inspection of the voucher individual (adult male) confirmed that it belongs to *N. melanospilus-*species group. This region is terra typica of *N. kwalensis*, a newly described cryptic species that can only be unambiguously diagnosed for female specimens [17], which were not available to us. While this apparently supports distinction of this lineage, a sample from adjacent population (KEN08–23, 15 km distant from KEN15–1 with no apparent dispersal barrier) contained three individuals with the widespread *N. melanospilus* haplotype (Ruvu) and morphologically, fish were typical *N. melanospilus*. Despite its relative distinctness, there was no statistical support to separate this haplotype. Clearly, more individuals and markers are needed to resolve validity of *N. kwalensis*. Our sampling (and laboratory analyses) were completed prior to the description of *N. kwalensis* and *N. prognathus* as separate species and we have no other specimens to further elaborate on our tentative findings. We acknowledge that this leaves the question of validity of *N. kwalensis*, and its geographical distribution, unresolved. Given the presence of the Ruvu lineage of *N. melanospilus* in coastal Kenya, it is possible that *N. melanospilus* sensu stricto rather than *N. kwalensis* may inhabit coastal plains of the northern Tanzania.

Regardless the taxonomic implications, we conclude that current *N. melanospilus* species group populations have been separated into at least five refugia that are consistent with divisions into the main river basins in the region. The lineage from at least one refugium (Ruvu) undergoes recent expansion and coexists with at least two other *N. melanospilus* lineages. Importantly, the only mitochondrial lineage (Rufiji) with a significant support for its genetic distinctiveness from other *N. melanospilus*-species-group lineages coexist widely with the Ruvu lineage and nuclear markers did not indicate the lack of panmixia. The fact that two lineages that were formally described to represent cryptic species of the complex – from the Wami (*N. prognathus*) and Ramisi (*N. kwalensis*) – are less distinct than the Rufiji lineage apparently supports the arguments of Wildekamp [39], who regarded minor morphological differences between *N. melanospilus* sensu stricto and the two putative cryptic species as normal intraspecific variation commonly seen in this [39] and many other *Nothobranchius* species [16, 40]. We acknowledge, however, that our results do not contradict existence of the two cryptic species either.

Intra-specific structure derived from microsatellite markers was largely congruent with mitochondrial data. Given a low number of individuals available, we have not genotyped Kenyan populations on microsatellite markers. Several populations that were not differentiated on mitochondrial data formed separate clusters at finer genetic substructuring. Those populations were typically genetically depauperated (low *H*_*e*_, *H*_*o*_ and *AR* estimates), suggesting that their distinct population genetic signatures arose from genetic drift due to either population bottlenecks or founder effects.

The populations were principally structured by their respective river basins. Main channels of large rivers did not constitute apparent barriers to dispersal in *N. melanospilus* this species group, in contrast to annual killifishes in particularly dry regions of Africa [15]. For example, two populations inhabiting the opposite banks of the lower Ruvu (T64 and TZN 17–9) had negligible (and non-significant) *F*_ST_ value (0.0042). Other adjacent populations had non-significant *F*_ST_ values, especially in the lower Ruvu (Additional file 3), but also in the Wami basin (7 km distant T09 and T83 in Tendigo swamp; *F*_ST_ = − 0.012) and, unexpectedly, between Ruvu (T14) and Rufiji (T17) populations (distance 164 km, *F*_ST_ = − 0.008). This corroborates that dispersal in equatorial region of coastal Tanzania might be more suitable for frequent dispersal across main river channel and among adjacent populations. The region experiences much longer duration of the wet phase, with two rainy seasons each year and a longer duration of habitat inundation compared to a single rainy season in subtropical Mozambique [41] with very brief periods of inundation [42, 43]. It demonstrates that *Nothobranchius* fishes experience variable climatic and ecological challenges that may affect their dispersal, diversification and coexistence in local killifish assemblages [44].

Our data on *Nothobranchius melanospilus* species group are largely congruent with phylogeographic patterns of other cyprinodontid fishes. For example, *Rivulus cylindraceus* from Cuba has wide distribution, with two haplogroups coexisting in some adjacent drainages and highly divergent haplogroups in isolated edges of the range [45]. In *Fundulus olivaceus* from midwestern and southern USA, distinctive haplogroups coexist in secondary contacts across drainages, while in sympatric *Fundulus notatus* four mitochondrial haplogroups are strictly isolated by respective river basins [46]. Finally, geographically widespread mummichog killifish, *Fundulus heteroclitus* from Atlantic coast of North America combines latitudinal isolation-by-distance pattern with a division into two sharply separated clades [47].

Within annual killifish, genetic variability of Neotropical annual killifish from the genus *Austrolebias* is also not structured by main river channels [46]. Especially lowland parts of major basins have been strongly affected by repeated marine transgressions and regressions during late Pliocene and Pleistocene [48]. For example, *Austrolebias bellottii* species group is widespread throughout the lower Paraná/La Plata and Uruguay basins, in a situation very similar to the *N. melanospilus* species group distribution pattern. A phylogeographic pattern of *A. bellottii* demonstrates repeated vicariance and dispersal events resulting in broad coexistence of major haplogroups across major river basins [48]. Lowland coastal areas of East Africa also commonly experienced repeated Quaternary marine regression and transgressions due to sea level changes [49] and climatic variability [50, 51], including recent connection between island of Zanzibar (where *N. melanospilus* is also present) and mainland [17].

We propose that distribution of *N. melanospilus* lineages was shaped by repeated marine regression and transgressions in the Quaternary [49–51], transverse faulting in the area that pertains to Holocene [55] as well as repeated rainfall pattern changes that led to switches between woodland savanna and semi-deciduous forest habitats in lowland areas of coastal Tanzania [53]. While such repeated fluctuations led to coexistence of formerly more restricted lineages in coastal areas, other lineages were left more isolated and might have evolved into evolutionary independent units. This situation is reminiscent to the population genetic structure of a tigerfish, *Hydrocynus tanzaniae*, with lineages of Middle Pleistocene-dated divergence between the Ruvu and Rufiji basins [54], as well as to examples from other continents, such as intraspecific divergences of a freshwater goby, *Rhinogobius duospilus*, in Hong Kong streams and Iberian cyprinid, *Squalius valentinus*, whose limited dispersal capabilities resulted in a clear intra-specific signature of Quaternary climatic oscillations [55, 56].

*Nothobranchius* fishes typically live in ephemeral pools [16]. However, across extensive range of the genus their habitats vary greatly in their size, connectivity and inundation patterns. *Nothobranchius* populations are finely structured in small, short-existing pools in dry inland region of southern Mozambique [14, 42] where main river channels form significant barriers to dispersal and lead to allopatric species and strong intra-specific diversification [15]. In contrast, humid equatorial region appears to enable greater dispersal across river channels and between river basins ([5, 10, 44], present study), with *Nothobranchius* fishes occurring in extensive marshes and semi-permanent streams [5, 16, 57]. *Nothobranchius* fishes are also present in the elevated part of equatorial East Africa (> 800 masl) and it remains to be tested how local populations are structured there. In that region, local topography and geographic history do not support as frequent dispersal as in coastal equatorial regions [10], while precipitation totals and existence of two rainy seasons differ from dry subtropical part of the genus distribution.

## Conclusions

Distribution of genetic lineages of annual fishes from a wet part of the genus distribution (tropical lowland) appears not to be constrained by dispersal limits posed by main river channels and closely related lineages frequently coexist in secondary contact zones. Annual fishes are promising research system for understanding links between ecological and evolutionary processes [4, 58] and research on their interspecific and intraspecific diversification promises to shed more light on complex issues of African biogeography [51, 59, 60].

## Supplementary information

**Additional file 1: **Protocols for genotyping of microsatellites and mitochondrial DNA. **Table S1.** Used microsatellite loci. **Table S2.** Primer sequences and marker features in GRZ strain of *N. furzeri*

**Additional file 2: **Additional figures supplementing results. **Figure S1.** Bayesian reconstruction of mitochondrial phylogeny of the *N. melanospilus* species complex based on 83 ingroup and 1 outgroup haplotypes of the 657 bp fragment of mitochondrial gene COI, including identity of individual samples. Bayesian inference posterior probabilities from MrBayes 3.2.6 are shown for each node. **Figure S2.** Correlation between Ln (distance) and linearized pairwise *F*_ST_ENA values (*F*_ST_/(1 − *F*_ST_)) tested by the Mantel tests (1000 permutations) analysed in GENEPOP. **Figure S3.** Mismatch distribution for two widespread mtDNA lineages. Dashed lines connect observed values and solid lines show the expected distribution under a demographic expansion model

**Additional file 3: **Additional table supplementing results. **Table S3.** Pairwise *F*_ST_ estimates based on 10 microsatellite loci

**Additional file 4: **Evaluation of 20 runs in STRUCTURE 2.3.4 (Hubisz et al. 2009) for each number of presumable clusters from K = 1 to K = 10. **Figure S2.** Likelihood (ln Pr(X|K)) of models in STRUCTURE for increasing number of hypothetical populations (K). **Figure S3.** Estimation of the best K division using the ΔK criterion according to Evanno et al. (2005). The values indicate relative increase of credibility depending on the number of K.

## Data Availability

The datasets supporting the conclusions of this article are included within the article and its Additional files 1, 2, 3, 4. Microsatellite data were deposited to Figshare (doi: 10.6084/m9.figshare.9631907). New sequences used in this study are available in GenBank (accession numbers MN413245–MN413327).

## Abbreviations

AR: Allelic richness
COI: The mitochondrial gene of cytochrome oxidase subunit 1
FDR: The false discovery rate approach
HWE: Hardy-Weinberg equilibrium
