# Supplementary material for: Genetic diversity of a widespread annual killifish from coastal Tanzania
Source: BMC Evol Biol. 2020 Jan 6;20:1. doi: 10.1186/s12862-019-1549-2 (PMC6943906; doi:10.1186/s12862-019-1549-2)
Supplement: Supplementary file 1 — Additional file 1: Protocols for genotyping of microsatellites and mitochondrial DNA. Table S1. Used microsatellite loci. Table S2. Primer sequences and marker features in GRZ strain of N. furzeri [file 12862_2019_1549_MOESM1_ESM.doc]

**Additional file 1** Protocols for genotyping of microsatellites and mitochondrial DNA.

(a) Genotyping of microsatellites

Microsatellite markers were developed previously using the *N. furzeri* genomic sequence (~10 Mb, for details on genomic sequencing see Reichwald et al. (2009)). The reaction mixture contained 1 µL extracted DNA, 5 µL PCR Multiplex Kit (Qiagen), primers of varying concentration (Table 1) and ddH_2_O to total volume 10 µL.

**Table S1: Used microsatellite loci**

| Marker-ID | Label | µM | Set | A | NA(%) |
| --- | --- | --- | --- | --- | --- |
| **Nfu_0006_FLI** | FAM | 0.2 | 1 | 38 | 6.96 |
| **Nfu_0009_FLI** | VIC | 0.2 | 1 | 12 | 10.20 |
| **Nfu_0012_FLI** | PET | 0.15 | 1 | 14 | 4.36 |
| **Nfu_0016_FLI** | VIC | 0.15 | 3 | 35 | 1.60 |
| **Nfu_0020_FLI** | NED | 0.5 | 4 | 57 | 0.90 |
| **Nfu_0027_FLI** | VIC | 0.1 | 4 | 73 | 13.59 |
| **Nfu_0029_FLI** | PET | 0.2 | 2 | 35 | 11.76 |
| **Nfu_0030_FLI** | FAM | 0.08 | 2 | 43 | 1.29 |
| **Nfu_0038_FLI** | NED | 0.2 | 3 | 34 | 1.93 |
| **Nfu_0140_FLI** | NED | 0.1 | 2 | 19 | 5.68 |

µM: final concentration of each forward and reverse primer in a multiplex PCR

Set: number of multiplex PCR set

A: number of alleles

NA(%): the average proportion of null alleles found in FreeNA

**Table S2: Primer sequences and marker features in GRZ strain of *N. furzeri***

| **Marker ID** | **Linkage group**  **in GRZ*** | **[repeat motif] no. of**  **repeat units in GRZ** | **Forward Oligo Sequence** | **Reverse Oligo Sequence** | **PCR product size in GRZ [bp]** |
| --- | --- | --- | --- | --- | --- |
| Nfu_0006_FLI | 12 | [AC]39 | GCAAGCAGCACCCTTTATTTC | GAAAGTAGGGGTCCCACACA | 281 |
| Nfu_0009_FLI | 17 | [GT]23 | GAGCGGGGACAAAGGTTTG | GGAGTTTTCCCCTTTTCAGG | 174 |
| Nfu_0012_FLI | 15 | [AC]22 | GATGCCACGCAGATAAAC | CATCTGTGTTTAGGCTGGTC | 185 |
| Nfu_0016_FLI | 20 | [GT]34 | TGCTGGCATCATCACTTCAC | GAAGAATGGCTTGTGGGAGT | 227 |
| Nfu_0020_FLI | 13 | [AC]36 | CTCGTTGTACTTCCAAGAGG | TACCGCTTTGGCTAAACACC | 233 |
| Nfu_0027_FLI | 16 | [AC]38 | GGACCACAGAGCAAAAGGAG | AGTTTTTGCCCCACTGTACG | 219 |
| Nfu_0029_FLI | 10 | [AC]18 | GTTTGAAAACCCACAATGCAC | CGCACATCTGATCACTCTCA | 221 |
| Nfu_0030_FLI | 11 | [GT]26 | CAGAAGCTAAAGGCCAGACG | GGGAAACAATAGGGAACCAC | 188 |
| Nfu_0038_FLI | 07 | [AC]26 | CAGTAGGAGGGAGAAGCAG | CTTTGTCAGCTTGCTCTAGG | 227 |
| Nfu_0140_FLI | 19 | [AC]13 | TGTTTACGCGAGTGATGG | GATGTTCTGATGTGGGTCAG | 187 |

* Linkage group is based on second-generation linkage map of GRZ strain of *N. furzeri* from Kirschner et al. (2012)

Temperature profile of PCR for microsatellites:

95°C (15 min) - 1 cycle

94°C (30 s) 56°C (90 s) 72°C (60 s) - 35 cycles

60°C (30 min) - 1 cycle

**Fragment Analysis**

The PCR products (1 µL) were added to a denaturing mixture of size standard (Genescan®, LIZ500, Applied Biosystems) and formamide. After 5 min denaturation at 96°C and 2 min cooling on ice, the mix was run on the ABI Prism® 3130 Genetic Analyzer (Applied Biosystems). DNA fragments were analysed using GeneMapper® v. 3.7 (Applied Biosystems).

(b) Genotyping of mitochondrial DNA

Partial mitochondrial *COI* gene was amplified using primers TRNYF1 (AGG GAG TTA CAA TCC ACC ACT ATT T) and TRNSR1 (ATG GGG GTT CAA TTC CTT CCT TT), alternatively, and a forward primer COI852F (CTT TAT TGT TTG AGC CCA CCA CA) designed by Nagy et al. (2017).

Temperature profile of PCR for *COI:*

94°C (2 min) - 1 cycle

94°C (30 sec) 60°C (30 sec) 72°C (3 min) - 30 cycles

72°C (7 min) - 1 cycle

PCR products were sequenced from one side by the Sanger method using BigDye Terminators v. 3.1 chemistry (Applied Biosystems).

Unsuccessfully amplified primers of cytochrome *b:*

FW40 (GCA AAT GAC TCC CTA ATT GAC C)

REV1019 (CCT CCA ATT CAT GTT AGG GTG)

REV941 (GTT GTT TTG AGG TGT GGA GG)

FW33 (AGC CAA CTC ATC ACA GGG TT)

REV733 (TAA CTA AGG GGT TTG CGG GG)

FW145 (CTA TTC TTA GCY ATA CAT TAC ACC TC)

FW 165 (CAC CTC RGA YAT TTC AAC TG)

References cited:

Kirschner, J., Weber, D., Neuschl, C., Franke, A., Bottger, M., Zielke, L., Powalsky, E., Groth, M., Shagin, D., Petzold, A., Hartmann, N., Englert, C., Brockmann, G. A., Platzer, M., Cellerino, A., Reichwald, K., 2012. Mapping of quantitative trait loci controlling lifespan in the short-lived fish *Nothobranchius furzeri* - a new vertebrate model for age research. Aging cell 11, 252-261.

Nagy B., Watters B.R., van der Merwe P.D.W., Cotterill F.P.D., Bellstedt, D.U., 2017. Nothobranchius cooperi (Teleostei: Cyprinodontiformes): a new species of annual killifish from the Luapula River drainage, northern Zambia, Afr. J. Aquat. Sci. 42, 201-218.

Reichwald, K., Lauber, C., Nanda, I., Kirschner, J., Hartmann, N., Schories, S., Gausmann, U., Taudien, S., Schilhabel, M. B., Szafranski, K., Glöckner, G., Schmid, M., Cellerino, A., Schartl, M., Englert, C., Platzer, M., 2009. High tandem repeat content in the genome of the short-lived annual fish *Nothobranchius furzeri*: a new vertebrate model for aging research. Genome Biol. 10, R16.
