# Supplementary material for: Genetic diversity of a widespread annual killifish from coastal Tanzania
Source: BMC Evol Biol. 2020 Jan 6;20:1. doi: 10.1186/s12862-019-1549-2 (PMC6943906; doi:10.1186/s12862-019-1549-2)
Supplement: Supplementary file 2 — Additional file 2: Additional figures supplementing results. Figure S1. Bayesian reconstruction of mitochondrial phylogeny of the N. melanospilus species complex based on 83 ingroup and 1 outgroup haplotypes of the 657 bp fragment of mitochondrial gene COI, including identity of individual samples. Bayesian inference posterior probabilities from MrBayes 3.2.6 are shown for each node. Figure S2. Correlation between Ln (distance) and linearized pairwise FSTENA values (FST/(1 − FST)) tested by the Mantel tests (1000 permutations) analysed in GENEPOP. Figure S3. Mismatch distribution for two widespread mtDNA lineages. Dashed lines connect observed values and solid lines show the expected distribution under a demographic expansion model [file 12862_2019_1549_MOESM2_ESM.docx]

**Additional file 2**

**Figure S1** Bayesian reconstruction of mitochondrial phylogeny of the N. melanospilus species complex based on 83 ingroup and 1 outgroup haplotypes of the 657 bp fragment of mitochondrial gene COI, including identity of individual samples. Bayesian inference posterior probabilities from MrBayes 3.2.6 are shown for each node

**
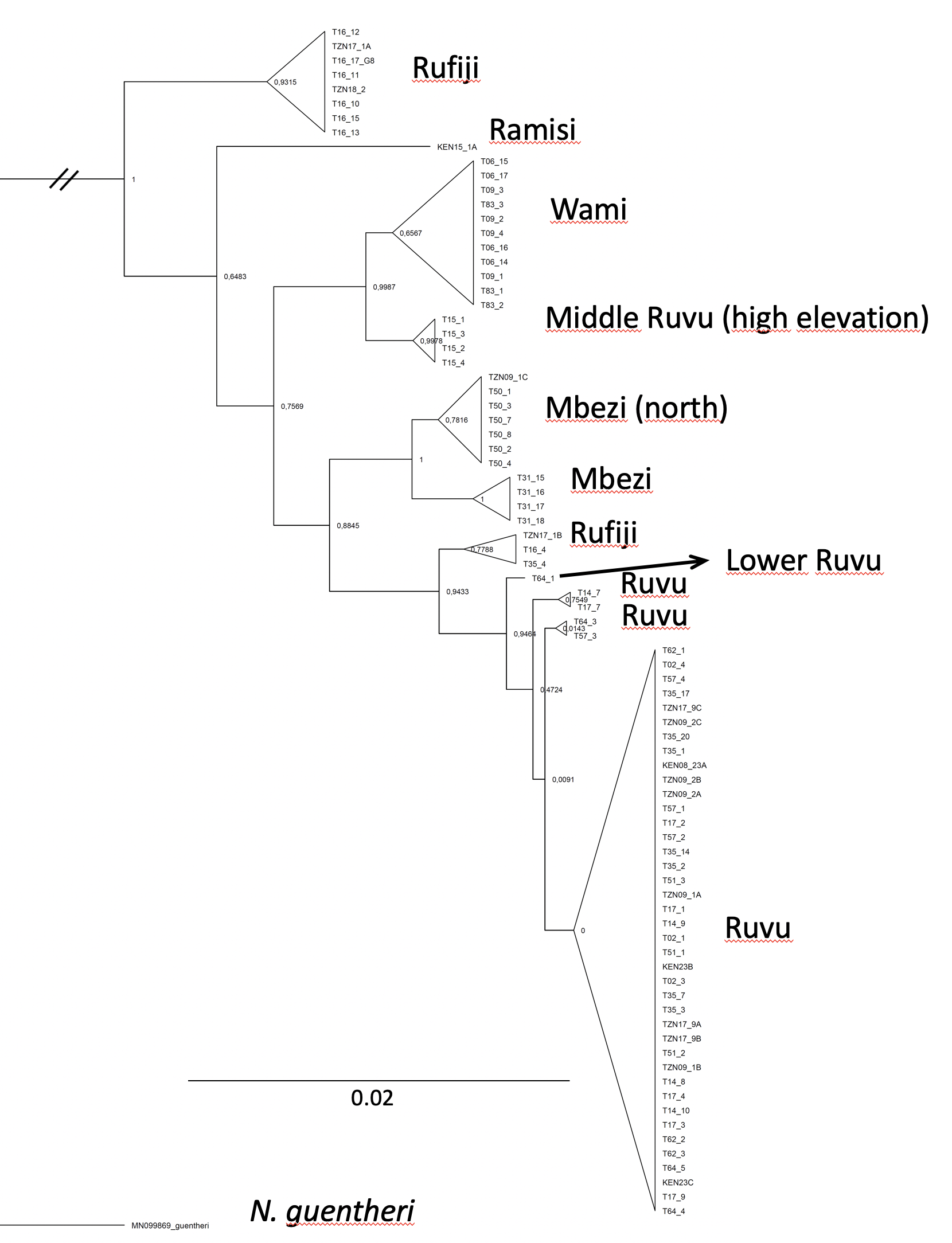
**

**Figure S2** Correlation between Ln (distance) and linearized pairwise F_ST_ENA values (FST /(1 − FST)) tested by the Mantel tests (1000 permutations) analysed in GENEPOP.

p=0.03

**Figure S3** Mismatch distribution for two widespread mtDNA lineages. Dashed lines connect observed values and solid lines show the expected distribution under a demographic expansion model.

(a) Ruvu (blue) group

**
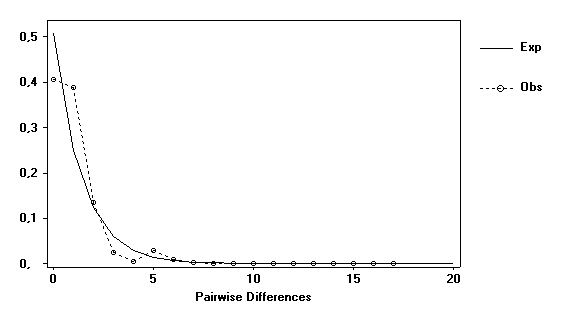
**

(b) Wami (orange) group

**
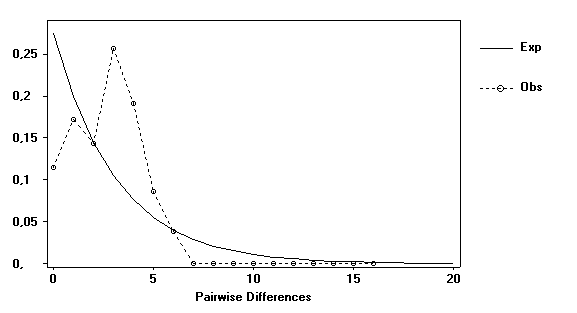
**
