# Supplementary material for: Genetic diversity of a widespread annual killifish from coastal Tanzania
Source: BMC Evol Biol. 2020 Jan 6;20:1. doi: 10.1186/s12862-019-1549-2 (PMC6943906; doi:10.1186/s12862-019-1549-2)
Supplement: Supplementary file 4 — Additional file 4: Evaluation of 20 runs in STRUCTURE 2.3.4 (Hubisz et al. 2009) for each number of presumable clusters from K = 1 to K = 10. Figure S2. Likelihood (ln Pr(X|K)) of models in STRUCTURE for increasing number of hypothetical populations (K). Figure S3. Estimation of the best K division using the ΔK criterion according to Evanno et al. (2005). The values indicate relative increase of credibility depending on the number of K. [file 12862_2019_1549_MOESM4_ESM.docx]

**Additional file 4** Evaluation of 20 runs in STRUCTURE 2.3.4 (Hubisz et al., 2009) for each number of presumable clusters from K=1 to K=10.

Figure S2: Likelihood (ln Pr(X|K)) of models in STRUCTURE for increasing number of hypothetical populations (*K*)


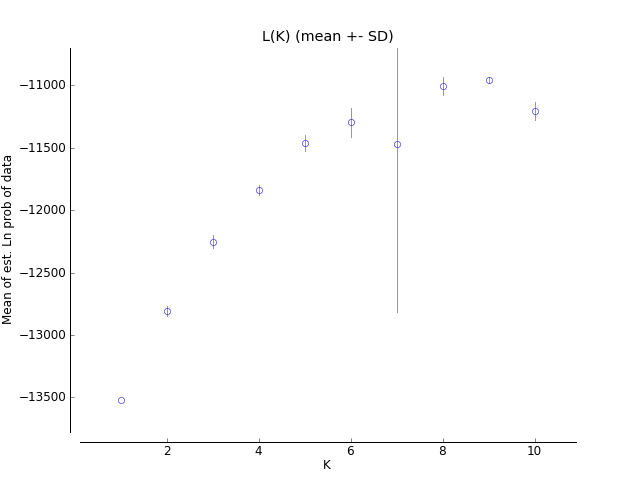


Figure S3 Estimation of the best *K* division using the ΔK criterion according to Evanno et al. (2005). The values indicate relative increase of credibility depending on the number of *K*.


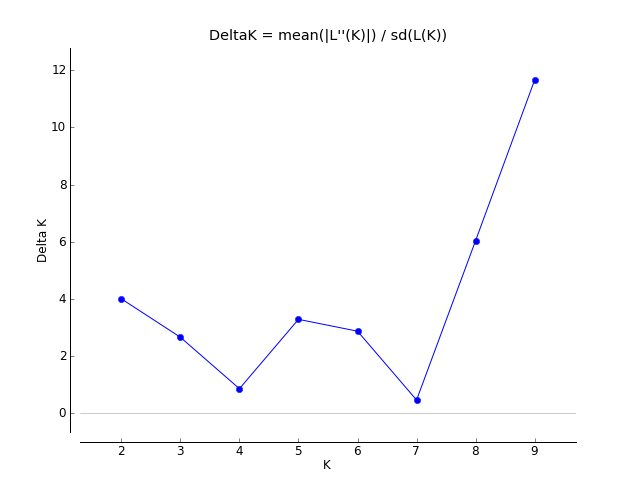


References cited:

Evanno, G., Regnaut, S., Goudet, J., 2005. Detecting the number of clusters of individuals using the software structure: a simulation study. Mol. Ecol. 14, 2611–2620.

Hubisz, M.J., Falush, D., Stephens, M., Pritchard, J.K., 2009. Inferring weak population structure with the assistance of sample group information. Mol. Ecol. Resour. 9, 1322–1332.
